# An update on the distribution, bionomics, and insecticide susceptibility of *Anopheles stephensi* in Ethiopia, 2018–2020

**DOI:** 10.1186/s12936-021-03801-3

**Published:** 2021-06-09

**Authors:** Meshesha Balkew, Peter Mumba, Gedeon Yohannes, Ephrem Abiy, Dejene Getachew, Solomon Yared, Amha Worku, Araya Gebresilassie, Fitsum G. Tadesse, Endalamaw Gadisa, Endashaw Esayas, Temesgen Ashine, Desta Ejeta, Sisay Dugassa, Mekonnen Yohannes, Wossenseged Lemma, Delenasaw Yewhalaw, Sheleme Chibsa, Hiwot Teka, Matt Murphy, Melissa Yoshimizu, Dereje Dengela, Sarah Zohdy, Seth Irish

**Affiliations:** 1 Abt Associates, PMI VectorLink Ethiopia Project, Addis Ababa, Ethiopia; 2 Dire Dawa University, Dire Dawa, Ethiopia; 3 Jigjiga University, Jigjiga, Ethiopia; 4 Addis Ababa University, Addis Ababa, Ethiopia; 5 Armauer Hansen Research Institute, Addis Ababa, Ethiopia; 6 Assosa University, Assosa, Ethiopia; 7 Mekelle University, Mekelle, Ethiopia; 8 Gondar University, Gondar, Ethiopia; 9 Jimma University, Jimma, Ethiopia; 10 US President’s Malaria Initiative (PMI), Addis Ababa, Ethiopia; 11 United States Agency for International Development (USAID), Addis Ababa, Ethiopia; 12 Malaria Branch, Division of Parasitic Diseases and Malaria, Center for Global Health, Centers for Disease Control and Prevention, Atlanta, GA USA; 13 US President’s Malaria Initiative, USAID, Washington, DC USA; 14 Abt Associates, PMI VectorLink Project, Rockville, MD USA; 15 Entomology Branch Division of Parasitic Diseases and Malaria, Center for Global Health, Centers for Disease Control and Prevention, Atlanta, GA USA

## Abstract

**Background:**

*Anopheles stephensi*, an invasive malaria vector, was first detected in Africa nearly 10 years ago. After the initial finding in Djibouti, it has subsequently been found in Ethiopia, Sudan and Somalia. To better inform policies and vector control decisions, it is important to understand the distribution, bionomics, insecticide susceptibility, and transmission potential of *An. stephensi*. These aspects were studied as part of routine entomological monitoring in Ethiopia between 2018 and 2020.

**Methods:**

Adult mosquitoes were collected using human landing collections, pyrethrum spray catches, CDC light traps, animal-baited tent traps, resting boxes, and manual aspiration from animal shelters. Larvae were collected using hand-held dippers. The source of blood in blood-fed mosquitoes and the presence of sporozoites was assessed through enzyme-linked immunosorbent assays (ELISA). Insecticide susceptibility was assessed for pyrethroids, organophosphates and carbamates.

**Results:**

Adult *An. stephensi* were collected with aspiration, black resting boxes, and animal-baited traps collecting the highest numbers of mosquitoes. Although sampling efforts were geographically widespread, *An. stephensi* larvae were collected in urban and rural sites in eastern Ethiopia, but *An. stephensi* larvae were not found in western Ethiopian sites. Blood-meal analysis revealed a high proportion of blood meals that were taken from goats, and only a small proportion from humans. *Plasmodium vivax* was detected in wild-collected *An. stephensi*. High levels of insecticide resistance were detected to pyrethroids, carbamates and organophosphates. Pre-exposure to piperonyl butoxide increased susceptibility to pyrethroids. Larvae were found to be susceptible to temephos.

**Conclusions:**

Understanding the bionomics, insecticide susceptibility and distribution of *An. stephensi* will improve the quality of a national response in Ethiopia and provide additional information on populations of this invasive species in Africa. Further work is needed to understand the role that *An. stephensi* will have in *Plasmodium* transmission and malaria case incidence. While additional data are being collected, national programmes can use the available data to formulate and operationalize national strategies against the threat of *An. stephensi*.

**Supplementary Information:**

The online version contains supplementary material available at 10.1186/s12936-021-03801-3.

## Background

*Anopheles stephensi* is one of the primary vectors of malaria in Asia [1]. In 2012, *An. stephensi* was found in Djibouti, marking the first confirmed report of this malaria vector from the African continent (earlier reports of *An. stephensi* in Egypt were later determined to be *Anopheles ainshamsi*) [2, 3]. In 2016, *An. stephensi* was found in the Somali Region in eastern Ethiopia [4]. Since then, *An. stephensi* has been found in an increasing number of sites in eastern Ethiopia [5], Sudan [9] and Somalia [9].

The spread of this vector is a grave concern for malaria control and elimination in the Horn of Africa, as data from Djibouti indicate the presence of *An. stephensi* has been associated with dramatic increases in malaria cases [6]. Suspected and confirmed malaria cases in Djibouti have increased nearly 30-fold, from 1684 in 2012 to 49,402 in 2019 [9]. While similar increases have not been yet reported in Ethiopia, recent work has shown that *An. stephensi* is a competent vector of *Plasmodium vivax* [7]. While *Anopheles arabiensis* remains the primary vector and *Anopheles pharoensis*, *Anopheles funestus* and *Anopheles nili* as secondary vectors of malaria in Ethiopia, the threat of the spread of *An. stephensi*, occupying a different ecological niche, is a major concern.

To improve understanding of the spread of *An. stephensi* in Ethiopia, regular sampling was conducted from 2018 to 2020. In addition, the bionomics and insecticide resistance status of *An. stephensi* was studied through routine surveillance and insecticide resistance monitoring activities. While some collection data from 2018 has been presented elsewhere [5], subsequent results are presented here, with the primary aim to guide the Ethiopian National Malaria Elimination Programme (NMEP) in implementing effective vector surveillance and control measures against this invasive mosquito species.

## Methods

### Study sites

**Fig. 1:**
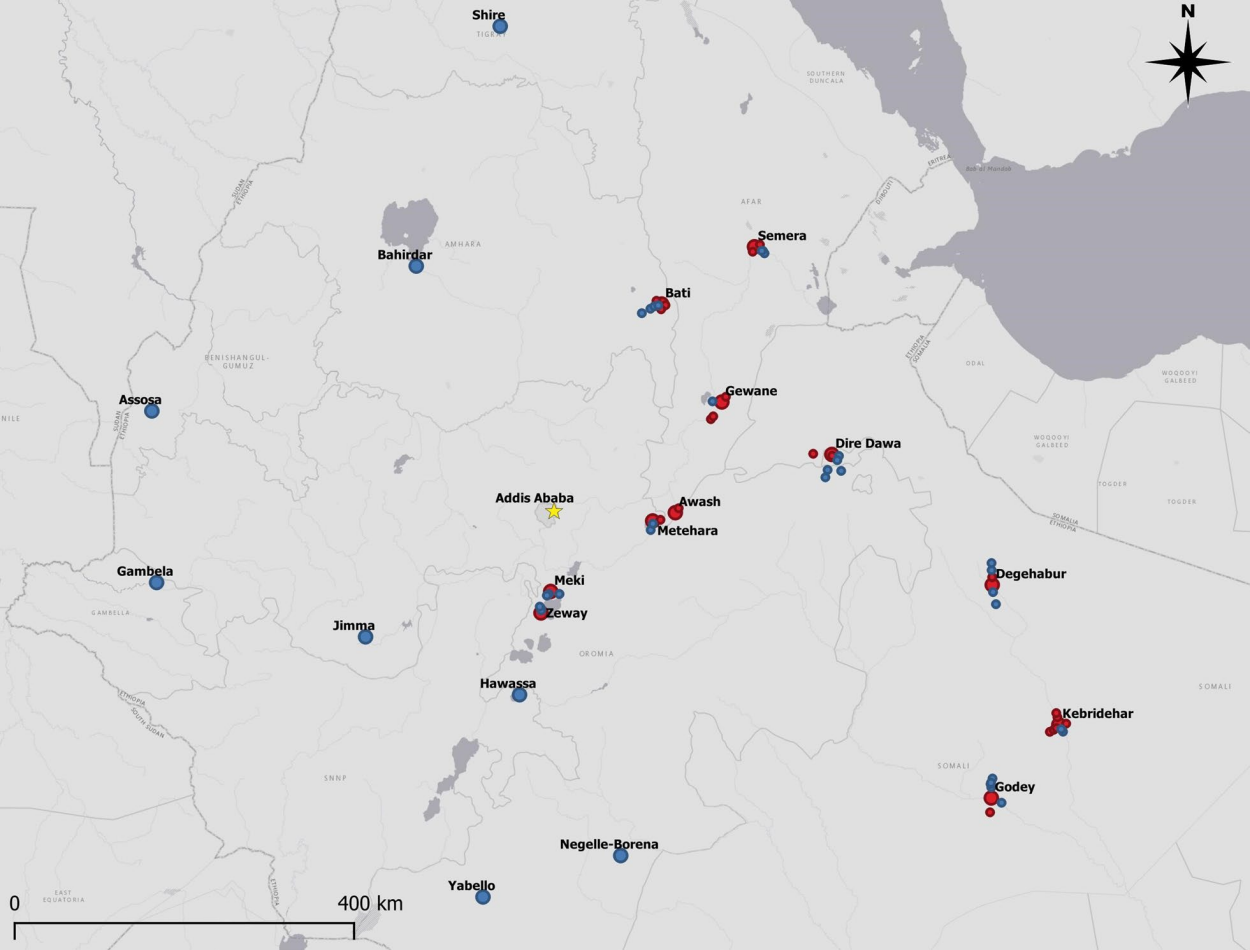
Sites positive (red) and negative (blue) for *Anopheles stephensi* in 2019 and 2020

In order to determine the distribution of *An. stephensi*, field surveys using one-time larval collections and identification of adults from reared larvae were conducted in 21 urban sites in Ethiopia in 2018 and 2019. In 2020, field surveys were expanded into peri-urban and rural sites within 20-km radius of 11 urban areas where *An. stephensi* had previously been collected. Adult mosquito collections were made in 10 sites in 2018, and 4 sites in 2019 and 2020. All sites where *An. stephensi* surveys were conducted are shown in Fig. 1 and described in Table 1.

**Table 1:** Sites sampled for larval and adult *Anopheles stephensi* in 2018–2020

Site	GPS coordinates	*Anopheles stephensi* present					
		2018		2019		2020	
		Larval collections	Adult collections	Larval collections	Adult collections	Larval collections	Adult collections
Assosa	10.062880, 34.543805			−			
Awash Sebat Kilo	8.988937, 40.160936	+	+	+	+	Urban (+)/rural (+)	+
Bahirdar	11.591264, 37.381047			−			
Bati	11.191347, 40.014825	+	+			Rural (+)	
Degehabur	8.223978, 43.558388	+	+			Rural (+)	
Dire Dawa	9.602669, 41.840532	+	+	+	+	Urban (+)/rural (+)	+
Erer Gota	9.555372, 41.384327	+	+				
Gambela	8.247653, 34.594831			−			
Gewane	10.157669, 40.660508	+	+	+		Rural (+)	
Godey	5.952589, 43.556624	+	+			Urban (+)/rural (+)	
Hawassa	7.053381, 38.489377			−			
Jigjiga	9.353974, 42.795313	+	+				
Jimma	7.669907, 36.837115			−			
Kebridehar	6.734321, 44.276404	+	+		+	Rural (+)	+
Meki	8.152866, 38.823858			+		Urban (+)/rural (−)	
Metehara	8.901551, 39.917774			+	+	Urban (+)/rural (+)	+
Negelle-Borena	5.336451, 39.575286			−			
Semera	11.792397, 41.010032	+	+	+		Rural (+)	
Shire	14.101822, 38.28188			−			
Yabello	4.893769, 38.097239			−			
Zeway	7.924096, 38.719499			+		Urban (+)/rural (−)	

Collections that found *An. stephensi* are designated with a “+” and collections that were performed, but that did not find *An. stephensi* are designated with a “−”

### Mosquito collection

#### Collection of larvae and pupae

Larvae and pupae were sampled in each site through a survey conducted by a team of three collectors who inspected the urban areas on foot, sampling all visible bodies of standing water and water-holding containers. Surveys generally lasted 6–7 days per site; all mosquito larvae collected were reared to adults for identification using a morphological key [8]. The survey teams were guided by staff with local knowledge of the area; surveys were not systematically conducted. GPS points of survey sites were not recorded until 2020. In 2020, to investigate whether *An. stephensi* had spread outside of urban areas into peri-urban and rural areas, larval collections were made in peri-urban areas or villages within 20 km of urban areas where *An. stephensi* had been found. Additionally in 2020, the presence of *Aedes* larvae (generally *Aedes aegypti*) was also recorded in the surveyed sites. In addition to larvae collected to determine the distribution of *An. stephensi*, larvae were also collected and reared to adults for insecticide susceptibility tests in 2018 (two sites), 2019 (five sites), and 2020 (four sites).

#### Collection of adult mosquitoes

Longitudinal surveillance of adult *An. stephensi* took place in Dire Dawa and Kebridehar from June to December 2019 and in Awash Sebat Kilo and Metehara towns from August to December 2019 (rainy season). The following methods were used in each site each month: human landing collections (HLC) (6 indoor and 6 outdoor collection nights), pyrethrum spray catches (PSC) (20 houses), CDC light traps (12 indoors and 12 outdoors), animal-baited tent traps (3 nights), and manual aspiration from animal shelters (2–20 collections per site). Additionally, black resting boxes (6 nights) were placed outdoors in the same compounds of HLC houses in Dire Dawa and Kebridehar, and near a horse stable in Dire Dawa.

HLCs were conducted indoors and outdoors at the same houses each month between 18.00 and 06.00 h. Mosquito collectors caught mosquitoes using mouth aspirators and placed them in labelled paper cups covered with mosquito netting. All mosquitoes collected each hour were aspirated into the same paper cup. Each hour, the collectors swapped positions between indoors and outdoors. If collectors showed symptoms of malaria, they were referred to health centres for free consultation and treatment.

PSCs were conducted between 06.00 and 09.00 h. Any structural gaps in the house were blocked and any food or cooking utensils and domestic animals were removed from the house. White sheets were spread on the floor of each room inside the house and a commercially available pyrethroid aerosol spray was sprayed inside the house. The house was closed for 10 min and then the sheets were individually carried outside and inspected for knocked-down mosquitoes.

CDC light traps (Bioquip, Rancho Dominguez, CA, USA) were set each day between 16.00 and 19.00 h. The indoor traps were suspended at 1.5 m at the foot of a bed, with residents of the household sleeping under their own insecticide-treated nets (if nets were not available, they were provided). Mosquitoes were retrieved from each trap the following morning between 06.00 and 09.00 h. Outdoors, a temporary shelter at a distance of 10 m from the same house was constructed and a volunteer slept on a camp bed protected by a treated net. A trap was hung on a pole 1.5 m above the ground by the feet of the volunteer.

Animal-baited tent traps were composed of a tethered ox, cow or goat under an untreated tent raised off the ground by 5 cm to allow mosquitoes to enter. The animal was kept inside the tent from 18.00 and resting mosquitoes on the wall of the tent were collected the following morning between 06.00 and 07.00 h. Any mosquitoes present in the tent were collected with a mouth aspirator and put into a paper cup, covered with mosquito netting, until no more mosquitoes were found.

In 2019, manual aspiration was conducted using a mouth aspirator to collect mosquitoes resting in animal shelters, but in 2020 this activity was replaced by Prokopack aspirators following the President’s Malaria Initiative (PMI) COVID-19 mitigation measures. Generally, horse stables were composed of walls on two sides and fences on two sides, with a corrugated tin roof. Goat and cattle shelters were made of brick walls on all sides with either a corrugated tin or thatched roof. Aspiration was conducted in the shelters between 06.00 and 09.00 and each shelter was inspected for 10–15 min. Any mosquitoes present were aspirated with a mouth aspirator into a paper cup, covered with mosquito netting while the Prokopack collections were kept in collection cups.

Black resting boxes were constructed of cardboard boxes in which the interior was lined with black nylon cloth. The boxes were placed in the compound of houses assigned for HLCs and horse stables before 18.00 and were inspected for the presence of mosquitoes the following morning between 06.00 and 07.00. Any mosquitoes present in the boxes were collected with a mouth aspirator into a paper cup covered with mosquito netting, until no more mosquitoes were found.

*Anopheles* mosquitoes were identified morphologically to species using a key by Coetzee [8] and stored individually in Eppendorf tubes with silica gel for laboratory processing.

### Blood meal analysis

The abdomens of blood-fed mosquitoes collected in 2019 from Dire Dawa and Kebridehar were subjected to a direct enzyme-linked immunosorbent assay (ELISA) following the method described in Beier et al. [10]. Briefly, a homogenate of each specimen was prepared in 50 µL of phosphate buffer saline (PBS) and transferred into an individual well of a 96-well assay plate and incubated for 3 h. For each wash step, 200 µL of PBS-Tween (0.5% Tween 20 in PBS) was used. The wells were washed twice and then incubated with 50 µL of conjugate per well for 1 h. The conjugate was incubated for 3 h at 4 °C before use and consisted of host-specific peroxidase-labelled monoclonal antibody of human, bovine, goat or dog. Positive and negative controls included whole blood samples collected from each host and non-blood-fed insectary-reared *An. arabiensis*, respectively. The total volume in the wells was removed by aspiration, and the plate was washed three times and incubated with 100 µL ABTS for 30 min. Following incubation, absorbance was immediately measured using a spectrophotometer at 405 nm (ELX800, BioTek, Winooski, VT, USA).

### Detection of *Plasmodium* sporozoites

All adult mosquitoes collected during longitudinal monitoring in Dire Dawa and Kebridehar in 2019 were tested for presence of sporozoites, using circumsporozoite (CS) ELISA. The heads and thoraces from all morphologically identified *An. stephensi* were assayed to detect antibodies against the CS proteins of *Plasmodium* *falciparum*, *Plasmodium vivax*-210 (Pv-210), and *P. vivax*-247 (Pv-247), using the sandwich CS-ELISA according to the protocol established by Wirtz et al. [11]. At least 4 negative controls and 4 positive controls were used for each ELISA plate. The cut-off value for the CS-ELISA was determined as two times the mean absorbance value of negative samples. Positive samples were not boiled and retested.

### Insecticide susceptibility tests

Insecticide susceptibility tests were conducted on adult *An*. *stephensi* reared from wild larvae from two sites in 2018 (Dire Dawa, Kebridehar), five sites in 2019 (Awash Sebat Kilo, Dire Dawa, Gewane, Kebridehar, Semera), and four sites in 2020 (Awash Sebat Kilo, Godey, Meki, Metehara) following standard procedures [12]. Seventy-five to 100 mosquitoes from each population were tested for each insecticide and 50 were used for controls. The insecticides used were 0.1% bendiocarb, 0.1% propoxur, 0.25% pirimiphos-methyl, 0.05% alpha-cypermethrin, 0.05% deltamethrin, and 0.75% permethrin.

Larval susceptibility assays were conducted in November 2020 to determine the susceptibility of *An. stephensi* larvae to temephos, an organophosphate larvicide. Larvae from five sites (Awash Sebat Kilo, Dire Dawa, Kebridehar, Meki, Semera) were tested. Assays were conducted according to an established protocol [13]. Briefly, temephos was added to cups of tap water to produce 250-mL volumes of concentrations ranging from 0.125 to 0.00375 mg/L, to calculate the concentration killing 50% and 95% of larvae. The estimated diagnostic dose of 0.25 mg/L was used to indicate resistance [14]. Approximately 25 third-instar larvae of *An. stephensi* were added to cups and mortality was recorded 24 h later. Four cups were used per dose to achieve 100 larvae tested per dose. Larvae and pupae collected from the same habitat were raised to adults for species identification to confirm *An. stephensi*.

### Piperonyl butoxide synergist assays

In 2018, piperonyl butoxide (PBO) synergist assays were conducted on *An. stephensi* from Dire Dawa and Kebridehar against two pyrethroids (deltamethrin and permethrin). In 2019, PBO synergist assays were conducted against three pyrethroids (alpha-cypermethrin, deltamethrin, permethrin) in Dire Dawa and against deltamethrin in Awash Sebat Kilo. In 2020, synergist assays were conducted against the same three pyrethroid insecticides in Awash Sebat Kilo, Godey, Meki, and Metehara. The synergist assays were conducted by pre-exposing mosquitoes to a 4% PBO paper for 60 min. Mosquitoes were then transferred to tubes with the pyrethroid of interest for 60 min and the susceptibility was determined as described for adult susceptibility tests described above.

### Resistance intensity

In Awash Sebat Kilo (2019, 2020), Meki (2020) and Metehara (2020), the resistance intensity of *An. stephensi* to alpha-cypermethrin, deltamethrin and permethrin was assessed through exposure to 1×, 5× and 10× the diagnostic dose. Mosquitoes were exposed to the insecticides for 60 min, and susceptibility assessed according to procedures described above.

## Results

### Distribution of *Anopheles stephensi*

In 2019, *An. stephensi* were found in three of the 11 urban sites where larval surveillance was conducted (Fig. 1). In Meki, larvae were found in tyres, concrete water containers, water tanks, and discarded buckets. In Metehara, larvae were collected from water tanks. In Zeway, larvae were found in tyres, water drums and concrete water containers. Other *Anopheles* larvae collected included *Anopheles gambiae *sensu lato (*s.l*.), *Anopheles rhodesiensis*, and *Anopheles cinereus* (Table 2). *Anopheles stephensi* was not detected in the towns of Assosa, Bahirdar, Gambela, Hawassa, Jimma, Negelle-Borena, Shire, and Yabello.

**Table 2:** Larvae of *Anopheles stephensi* and other *Anopheles* species collected from various habitat types in selected urban sites in Ethiopia, August–December 2019

Urban site	Larval habitat type	Total *Anopheles* larvae collected	*An. stephensi*	*An. gambiae s.l*	*An. rhodesiensis*	*An. cinereus*
Negelle-Borena	Water containers	55	0	13	0	0
	Water tanks	66	0	11	0	1
	Stagnant water pools	211	0	132	0	0
Yabello	Water tanks	39	0	13	0	0
	Stagnant water pools	55	0	23	0	0
	Cement water reservoirs	194	0	90	15	0
Jimma	Rain pools and puddles	378	0	148	0	0
Gambela	Rain pools and puddles	143	0	61	0	0
Assosa	Discarded tyres	150	0	86	0	0
	Rain pools and puddles	1618	0	1266	0	0
	Natural habitats	710	0	531	0	0
Bahirdar	Tyres	1681	0	1213	0	0
	Stagnant water pools	807	0	294	0	0
Meki	Tyres	45	24	0	0	0
	Concrete water container	68	43	20	0	0
	Water tanks	36	19	10	0	0
	Discarded buckets	2	0	1	0	0
Zeway	Tyres	24	14	0	0	0
	Water drums	1	0	0	0	0
	Concrete water containers	12	3	5	0	0
Hawassa	Water drums	7	0	5	0	0
	Concrete water containers	6	0	4	0	0
	Waste bin	12	0	9	0	0
	Plastic bucket	4	0	4	0	0
Shire	Tyres	14	0	14	0	0
	Rain pools and puddles	2327	0	990	0	0
	Natural habitats	208	0	130	0	0
Metehara	Water tanks	1075	322	0	0	0

In 2020, to determine whether *An. stephensi* was present in rural areas, *kebeles* (rural and peri-urban villages) within 20 km of an urban site were searched for larvae. *Anopheles stephensi* was found in 21 of the 55 *kebeles* investigated. The results of these searches are shown in Table 3. Larval sites in which *An. stephensi* were found included: water drums, plastic water tanks, puddles, concrete wells, plastic sheets, discarded tyres, flooded cement floors of a house under construction, and metal water tanks (Additional file 1: Table S1). In 40% of the sites where *An. stephensi* were found, *Aedes* larvae were also collected (Table 3).

**Table 3:** Larval survey results of *Anopheles stephensi* and *Aedes* larvae in kebeles within 20 km of urban sites in Ethiopia where *Anopheles stephensi* had been found previously, 2020

Nearest town	Number of visited *kebeles*	Number of *kebeles* positive for *An. stephensi*	Number of potential larval sites inspected	Number of larval sites positive for *An. stephensi* (%)	Number of larval sites positive for *Aedes* (%)	Number of *An. stephensi* larval sites also containing *Aedes* larvae (%)
Awash	1	1	3	1 (33)	1 (33)	1 (100)
Bati	7	3	165	6 (4)	42 (25)	4 (67)
Degehabur	6	2	32	7 (22)	7 (22)	2 (29)
Dire Dawa	7	2	17	2 (12)	8 (47)	2 (100)
Gewane	4	3	127	10 (8)	24 (19)	7 (70)
Godey	6	1	24	1 (4)	6 (25)	0
Kebridehar	8	6	40	13 (33)	6 (15)	0
Meki	5	0	17	0	0	0
Metehara	3	1	12	1 (8)	2 (17)	0
Semera	5	2	136	3 (2)	22 (16)	2 (67)
Zeway	3	0	16	0	0	0
Total	55	21	589	44 (7)	118 (20)	18 (40)

### *Anopheles stephensi* in longitudinal surveillance sites

A total of 1040 adult *An. stephensi* were collected from Dire Dawa (n = 412), Kebridehar (n = 368), Awash Sebat Kilo (n = 154), and Metehara (n = 106) in 2019 (Table 4). The majority (n = 585, 56.3%) were collected in animal shelters (cattle, goats, sheep, horses) using manual aspiration. In the peri-urban areas of Dire Dawa, nearly 39% (n = 159) of *An. stephensi* collected were found resting in black boxes placed in the compounds of houses with horse stables. Black resting boxes were not effective in compounds without horse stables. Cattle-baited traps caught 19.0% (n = 198) of all *An. stephensi* collected. The most common mosquito sampling methods, PSC, HLC and CDC light traps, were less effective than aspiration, black boxes and animal-baited traps in the collection of adult *An. stephensi*. The greatest numbers of *An. stephensi* were collected in August, September and October.

**Table 4:** *Anopheles stephensi* collected in four longitudinal monitoring sites in Ethiopia in 2019 using different sampling methods

Month 2019	Dire Dawa							Kebridehar							Awash Sebat Kilo						Metehara					
	PSC	HLC	CDC light trap	Hand Collection	Black resting box	Cattle-baited Tent Trap	Total	PSC	HLC	CDC light trap	Hand Collection	Black resting box	Cattle-baited Tent Trap	Total	PSC	HLC	CDC light trap	Hand Collection	Cattle-baited Tent Trap	Total	PSC	HLC	CDC light trap	Hand Collection	Cattle-baited Tent Trap	Total
June	0	0	0	ND	0	ND	0	4	1	3	ND	0	ND	8	ND	ND	ND	ND	ND	ND	ND	ND	ND	ND	ND	ND
July	3	0	0	18	0	ND	21	1	0	0	0	0	ND	1	ND	ND	ND	ND	ND	ND	ND	ND	ND	ND	ND	ND
August	1	0	0	127	16	16	160	2	0	0	5	0	6	13	0	4	0	20	ND	24	1	4	0	2	ND	7
September	0	0	3	24	82	9	118	4	0	0	19	0	18	41	0	2	0	36	10	48	5	7	18	19	4	53
October	0	0	1	26	56	9	92	1	4	0	79	0	29	113	2	1	0	37	10	50	2	0	2	12	5	21
November	0	0	0	5	2	6	13	3	0	0	46	0	29	78	0	0	0	11	1	12	1	0	0	3	3	7
December	0	0	0	5	3	0	8	14	0	0	63	0	37	114	1	0	0	19	0	17	0	0	3	9	6	18
Total	4	0	4	205	159	40	412	29	5	3	212	0	119	368	3	7	0	123	21	154	9	11	23	45	18	106

Sampling methods used in each site each month included: HLC: 6 indoor and 6 outdoor collection nights; PSC: 20 houses; CDC light traps: 12 indoors and 12 outdoors; manual aspiration from animal shelters: 2–20 collections per site; black resting boxes: 6 nights; animal baited tent traps: each for 3 nights

### Blood meal identification

**Fig. 2:**
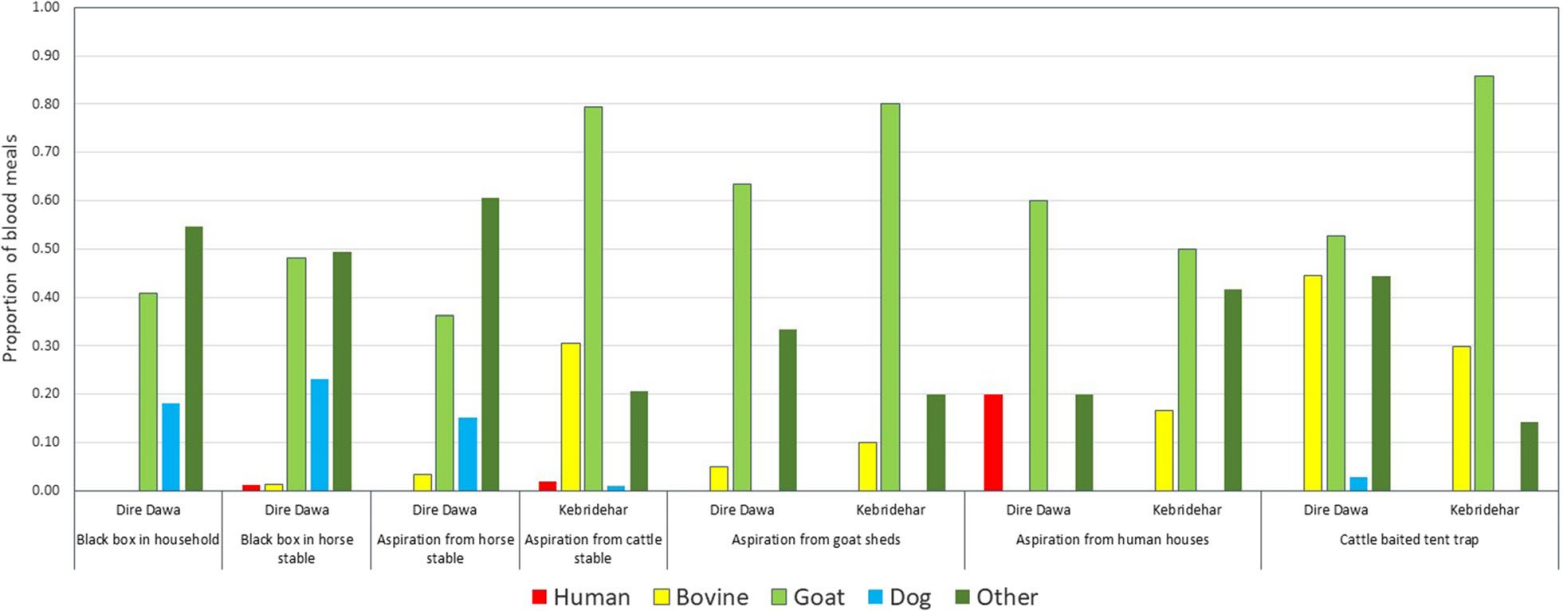
Identification of blood meal sources in adult *Anopheles stephensi* (2019) collected using different methods in Dire Dawa and Kebridehar, Ethiopia, 2019

A total of 631 visibly blood-fed *An. stephen*si from Dire Dawa and Kebridehar sites, collected in 2019, were tested by ELISA for blood meal sources. One (0.25%) of the 394 *An. stephensi* from Dire Dawa and 0/237 from Kebridehar were found with human blood only. In contrast, 29.7% and 53.2% were found to have fed on goats alone, and 1.02% and 0.4% on cows alone, in the respective sites. Dog blood alone was the source of 2.03% of blood meals of *An. stephensi* from Dire Dawa and 1.3% from Kebridehar. Mixed blood was found in 20.9% of *An. stephensi* tested, with 0.32% of these a mixture of human and goat blood, and 0.16% a mixture of human, bovine, goat, and dog. The remaining 38.4% of blood meals were not identified. The frequency of blood meals from each source is provided in Fig. 2.

### *Anopheles stephensi* infection with *Plasmodium falciparum* and *Plasmodium vivax* sporozoites

All of the 780 adult *An. stephensi* specimens (412 from Dire Dawa and 368 from Kebridehar) were tested for *P. falciparum* and *P. vivax* CS proteins. Of these, three were positive for *P. vivax*, with infection rates of 0.5% and 0.3% from Dire Dawa and Kebridehar, respectively. The two positive samples from Dire Dawa were of the Pv-210 variant and the single positive sample from Kebridehar was of the Pv-247 variant. None of the tested *An. stephensi* was positive for *P. falciparum*.

### Insecticide susceptibility

In 2018, in both Dire Dawa and Kebridehar, *An. stephensi* was found to be resistant to all pyrethroids and carbamates tested and was susceptible only to pirimiphos-methyl (Table 5). Pre-exposure of mosquitoes to PBO increased susceptibility of *An. stephensi* to deltamethrin to 96% in Dire Dawa. PBO pre-exposure fully restored susceptibility (100% mortality) to both deltamethrin and permethrin in Kebridehar.

**Table 5:** Susceptibility test results of *Anopheles stephensi* in diagnostic and synergist assays

Type of assay	Insecticide class	Insecticide	Concentration	Percentage mortality (number tested)										
				2018		2019					2020			
				Dire Dawa	Kebridehar	Dire Dawa	Kebridehar	Gewane	Semera	Awash Sebat Kilo	Awash Sebat Kilo	Meki	Metehara	Godey
Diagnostic dose	Pyrethroids	Permethrin	0.75%	79 (100)	78 (100)	86 (100)	76 (100)	68 (100)	67 (100)	39 (100)	43 (100)	72 (100)	93 (100)	10 (100)
		Deltamethrin	0.05%	54 (100)	80 (100)	64 (100)	74 (100)	31 (100)	37 (100)	68 (100)	15 (100)	59 (100)	17 (100)	7 (100)
		Alpha-cypermethrin	0.05%	82 (100)	80 (100)	30 (100)	64 (100)	20 (100)	51 (100)	69 (100)	10 (100)	33 (100)	62 (100)	1 (100)
	Carbamates	Bendiocarb	0.05%	19 (100)	73 (100)	4 (100)	17 (100)	60 (100)	57 (100)	5 (100)	13 (100)	4 (100)	7 (100)	20 (100)
		Propoxur	0.10%	77 (100)	68 (100)	59 (100)	38 (100)	77 (100)	99 (100)	73 (100)	19 (100)	9 (100)	17 (100)	79 (100)
	Organophosphates	Pirimiphos-methyl	0.25%	100 (100)	100 (100)	27 (100)	49 (100)	92 (100)	99 (100)	93 (100)	1 (100)	0 (100)	35 (100)	67 (100)
Synergist assays	Pyrethroids	Permethrin	0.75%		69 (75)	85 (75)					70 (75)	43 (75)	43 (75)	16 (75)
		Permethrin + PBO	0.75%/4%		100 (75)	100 (75)					100 (75)	100 (75)	100 (75)	100 (75)
		Deltamethrin	0.05%	45 (75)	49 (75)	67 (75)				61 (75)	21 (75)	57 (75)	39 (75)	4 (75)
		Deltamethrin + PBO	0.05%/4%	96 (75)	100 (75)	97 (75)				100 (100)	100 (75)	100 (75)	99 (75)	92 (75)
		Alpha-cypermethrin	0.05%			83 (75)					31 (75)	25 (75)	9 (75)	8 (75)
		Alpha-cypermethrin + PBO	0.05%/4%			100 (75)					93 (75)	100 (75)	100 (75)	96 (75)

In 2019, *An. stephensi* from all five sites were highly resistant to bendiocarb, alpha-cypermethrin, deltamethrin, and permethrin (Table 5). *Anopheles stephensi* were susceptible to propoxur and pirimiphos-methyl in only one site, Semera (99% mortality for both insecticides), and resistant to pirimiphos-methyl in Dire Dawa and Kebridehar. Possible resistance to pirimiphos-methyl was recorded in Awash Sebat Kilo and Gewane. In the synergist assays, pre-exposure to PBO restored full susceptibility to alpha-cypermethrin and permethrin in Dire Dawa and to deltamethrin in Awash Sebat Kilo, and substantially increased susceptibility to deltamethrin (up to 97% mortality) in Dire Dawa.

In 2020, *An. stephensi* resistance to bendiocarb, propoxur, pirimiphos-methyl, and the three pyrethroids (deltamethrin, permethrin, alpha-cypermethrin) was observed in the 4 sites tested (Table 5). When *An. stephensi* were pre-exposed to PBO before exposure to pyrethroids, susceptibility was fully restored to permethrin in all 4 sites, to deltamethrin in 3/4 sites, and to alpha-cypermethrin in 2/4 sites (Table 5).

**Fig. 3:**
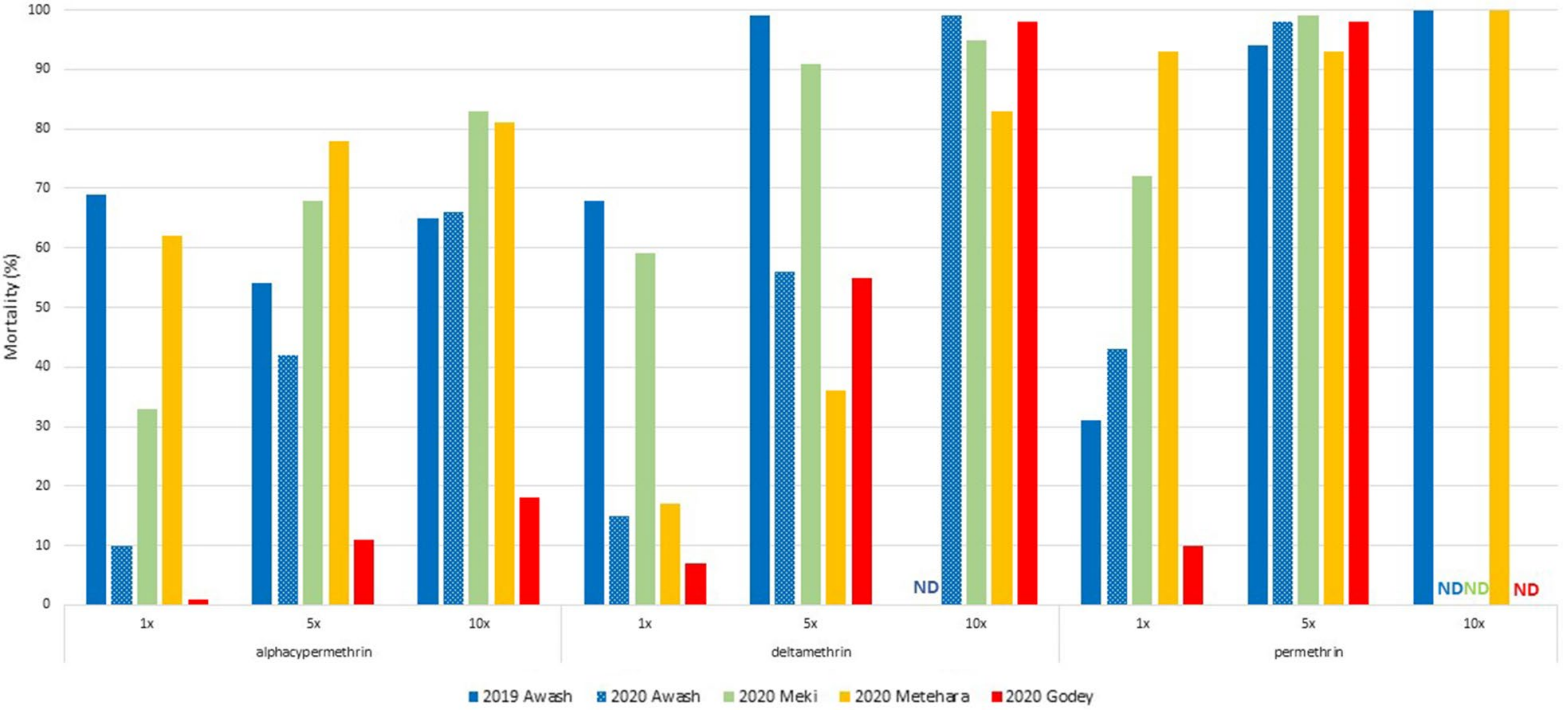
Intensity of resistance to pyrethroids in *Anopheles stephensi* in 2019 (Awash Sebat Kilo, designated Awash) and 2020 (Awash Sebat Kilo, Meki, Metehara, and Godey), Ethiopia. Tests were not done (ND) if susceptibility (> 98%) was attained with a lower dose

Resistance intensity in Awash Sebat Kilo in 2019 and in Awash Sebat Kilo, Meki, Metehara, and Godey in 2020 is shown in Fig. 3. At the diagnostic dose, resistance was found to all three pyrethroids in all locations and years, with the exception of Metehara in 2020, where possible resistance (93% mortality) was found to permethrin. Resistance to alpha-cypermethrin, even at 10× the diagnostic dose, was found in all sites and years. For deltamethrin, resistance or possible resistance was found at either the 5× level (Awash Sebat Kilo 2019 and Meki 2020) or 10× level (Awash Sebat Kilo 2020), however, even at 10× the diagnostic dose, *An. stephensi* in Metehara 2020 remained resistant to deltamethrin. Susceptibility to permethrin was found at 5× (Awash Sebat Kilo 2020 and Meki 2020) or 10× (Awash Sebat Kilo 2019 and Metehara 2020).

### Temephos susceptibility test results

All the *An. stephensi* populations tested were found to have 100% mortality at less than the threshold for susceptibility (0.25 mg/L). In Awash Sebat Kilo, Dire Dawa and Kebridehar, 100% mortality was observed at 0.125 mg/L. In Meki mortality was 100% at 0.03125 mg/L, and in Semera mortality was 100% at 0.0625 mg/L. The LC_50_ and LC_95_ values were calculated for 3 of the sites (Table 6).

**Table 6:** *Anopheles stephensi* lethal dose (LC) LC_50_ and LC_95_ values, with confidence intervals after exposure to temephos concentrations

Site	LC_50_ (95% CI) mg/L	LC_95_ (95% CI) mg/L
Dire Dawa	0.105 (0.099–0.109)	0.118 (0.114–0.113)
Kebridehar	0.019 (0.015–0.027)	0.031 (0.024–0.122)
Meki	0.012 (0.011–0.013)	0.025 (0.021–0.032)

## Discussion

Since the initial detection of *An. stephensi* in Ethiopia in 2016, efforts to map its distribution of *An. stephensi* have detected its presence in some parts of the country, but not in others. Surveys throughout Ethiopia in 2019 detected the presence of *An. stephensi* in three additional sites (Meki, Metehara, Zeway). These three sites lie within the distribution predicted by a spatial model of environmental suitability [15]. The surveyed sites that were negative for *An. stephensi* in western Ethiopia in 2019 do not prove the absence of *An. stephensi.* However, routine detection in sites in eastern Ethiopia and not in the western half of the country suggests that *An. stephensi* distribution may be limited. The limits of *An. stephensi* distribution are yet to be elucidated and are likely not to be static. Continued monitoring of *An. stephensi* populations in Ethiopia is necessary to understand the extent of their distribution and possible spread.

The larval sites where *An. stephensi* was found in 2019 and 2020 resemble those previously reported [5], such as water storage containers, barrels and wells. In addition, *An. stephensi* was found in puddles, wells and the flooded cement floor in a house under construction. In general, the percentage of inspected sites that were positive for *An. stephensi* was low (≤ 33%). The sites where *An. stephensi* were present often contained *Aedes* larvae, indicating that larval control of these sites might have benefits for prevention of both malaria and *Aedes*-borne diseases.

The highest numbers of adult *An. stephensi* were collected in the four longitudinal monitoring sites with manual aspiration of mosquitoes from animal shelters. Determining the most efficient collection method could not be done, unfortunately, as the number of collections made was not recorded. While the largest numbers of adult *An. stephensi* were collected in August, September and October, a more rigorous and standardized collection protocol is needed to determine patterns of seasonality. Furthermore, anecdotal reports indicate that *An. stephensi* may be present during the dry season. Determining the most effective collection method and the seasonality of *An. stephensi* remains a priority.

Blood meal analysis revealed high levels of zoophagy, particularly on goats; however, host blood meal from a large proportion of samples could not be identified through the ELISA method. This could be due to the blood meal host sources not being represented amongst the reagents used. Additionally, blood meal analysis using PCR could be used to identify species-specific blood meals [16]. Since many of the mosquitoes analysed in this study were collected from horse shelters, adding this host to the blood meal analysis activity is a priority. Nonetheless, the results from this work are in line with blood-feeding indices noted in India, where high levels of zoophagy were observed, even in urban settings [17].

In a previous study, *An. stephensi* collected in 2020 in Ethiopia were reared in the laboratory to determine vectorial capacity [7]. The findings suggested that Ethiopian *An. stephensi* are more competent vectors of *P. vivax* than *An. arabiensis*; however, little is known about sporozoite rates of wild *An. stephensi* in Ethiopia. In this study, *P. vivax* sporozoite rates of 0.5% and 0.3% were found in Dire Dawa and Kebridehar, respectively, though the percentage of human blood meals from the two locations was 0.25% and 0%, respectively. More work is needed to see whether collection bias may have resulted in underestimates of human feeding, or if the vector capacity of *An. stephensi* is efficient enough that even low levels of human feeding result in sporozoite rates similar to wild-caught *An. arabiensis* [7, 18].

Widespread pyrethroid resistance in *An. stephensi* has been reported from Asia [19] and resistance has also been reported in Ethiopia [20]. However, to combat and control this invasive vector, a fuller understanding of insecticide resistance profiles is necessary. While considerable variation was noted between years in the phenotypic susceptibility assay results, a general pattern of high levels of pyrethroid resistance was evident. Similarly, resistance to the carbamates propoxur and bendiocarb was noted. Resistance to pirimiphos-methyl was more variable, with susceptibility noted in some settings and high levels of resistance detected in others. Resistance to pyrethroids was intense for alpha-cypermethrin and deltamethrin, but less so for permethrin. This resistance appeared to be likely mediated in large part by oxidases, as pre-exposure of mosquitoes to PBO resulted in large increases of mortality.

The implications of resistance patterns are important for vector control decision making. Currently, insecticide treated nets (ITNs) and indoor residual spraying (IRS) is conducted in rural areas by the NMEP, but not in urban settings due to the documented low risk of malaria [21], resource limitations, and low community acceptance of IRS. New types of nets, such as PBO nets, may be useful vector control tools for use against *An. stephensi*. Further work is needed to understand *An. stephensi* susceptibility to chlorfenapyr and pyriproxyfen, additional insecticides used in bi-treated nets. IRS is largely conducted in rural areas using products containing pirimiphos-methyl and clothianidin, so further work is needed to clarify the susceptibility of *An. stephensi* to these insecticides as well.

Temephos has been used as a larvicide in Ethiopia to control *An. arabiensis*. All five sites where *An. stephensi* was tested for temephos susceptibility showed complete susceptibility. Further work is needed to determine the susceptibility to other larvicides that might be used for control of *An. stephensi* (e.g., *Bacillus thuringiensis* var. *israelensis*, pyriproxyfen).

While this work provides fundamental data on *An. stephensi* in Ethiopia, there were limitations. Firstly, with the exception of 2020, the larval surveys were not done according to a standardized grid-based protocol, that is, teams looked around the survey area and sampled any potential larval habitats as they were found. Only positive sites were recorded, and the number of dips per site and the GPS points of each site were not recorded. Furthermore, sites negative for *An. stephensi* were not recorded. This limits our understanding of the concentration of *An. stephensi* in the sites surveyed and future surveys will follow standard collection protocols that incorporate collection of these types of data. Secondly, large numbers of mosquitoes identified as *An. gambiae s.l*. were collected in tyres from Assosa, Bahirdar and Shire. Tyres are an uncommon larval site of *An. arabiensis*, and it would have been ideal to sequence some of these specimens to see if they were indeed *An. arabiensis*. However, the specimens were not kept after identification, so further collections will be needed to confirm this finding. Thirdly, the sporozoite rates were determined through CS ELISA. Reports of false positives have been found, particularly in cases when mosquitoes have fed on animals [22]. The recommendation to ensure that tests are true positives is to boil the samples at 100 °C for 10 min and retest. This was not done in this study, which is a source of concern, as most of the mosquitoes were collected from animal shelters and high animal feeding was noted. Further work is needed to provide additional confirmation of sporozoite-positive samples. Finally, there were occasionally large discrepancies between susceptibility test results, even when testing mosquitoes from the same location. For example, in Metehara in 2020, the first test with the diagnostic dose of alpha-cypermethrin resulted in a mortality of 62%, whereas a second test (conducted as part of the synergist work) found only 9%. While these did not change the interpretation of the test (that the population was resistant to alpha-cypermethrin), such wide variation could influence overall results. It is important to ensure to the extent possible that larvae collected from the field are reared, and adults tested, in standard conditions (feeding, temperature, larvae per pan, etc.) as much as possible [23].

More data are needed to determine the distribution, role as a vector of malaria parasites, and interventions that can effectively control *An. stephensi* in Ethiopia. This must be a priority not only for the NMEP in Ethiopia, but for the entire malaria control community. Sinka et al. [15] predicted that an additional 126 million people in Africa might be at risk of contracting malaria if *An. stephensi* spreads throughout Africa. While *An. stephensi* appears to be capable of colonizing urban, peri-urban and rural settings, malaria transmitted by urban *An. stephensi* might divert resources to urban settings at the expense of rural settings, where low health system capacity and longer distances to health services means the risk of dying from malaria is more likely.

## Conclusions

*Anopheles stephensi*, an invasive malaria vector in Africa, has been described as a potential threat to malaria control and elimination in Africa. First detected in 2016 in Ethiopia, *An. stephensi* now appears to be widespread, including in major urban and peri-urban areas, and remote rural areas along major transportation routes. Blood meal analysis showed that *An. stephensi* in Ethiopia were highly zoophagic, yet *P. vivax* sporozoite rates were likely higher than in the primary malaria vectors in Ethiopia, *An. arabiensis* and *An. pharoensis*, indicating potential to cause increases in malaria in urban areas. As vector control measures are considered, high levels of resistance to many of the insecticides used on ITNs and for IRS may render these interventions less effective [19], and therefore alternative interventions, such as new types of nets (PBO and bi-treated), and larviciding, may need to be considered.

## Supplementary Information

**Additional file 1: Table S1.** Larval collections made in 2020.

## Data Availability

All data generated are included in this manuscript and supplementary files.

## Abbreviations

CDC: Centers for Disease Control and Prevention
CS: Circumsporozoite
DNA: Deoxyribonucleic acid
ELISA: Enzyme-linked immunosorbent assay
HLC: Human landing collection
ITN: Insecticide-treated bed net
IRS: Indoor residual spraying
NMEP: National Malaria Elimination Programme
PBO: Piperonyl butoxide
PBS: Phosphate buffered saline
PCR: Polymerase chain reaction
PMI: U.S. President’s Malaria Initiative
PSC: Pyrethrum spray collection
