# Correction to: An update on the distribution, bionomics, and insecticide susceptibility of *Anopheles stephensi* in Ethiopia, 2018–2020

**DOI:** 10.1186/s12936-021-03852-6

**Published:** 2021-07-28

**Authors:** Meshesha Balkew, Peter Mumba, Gedeon Yohannes, Ephrem Abiy, Dejene Getachew, Solomon Yared, Amha Worku, Araya Gebresilassie, Fitsum G. Tadesse, Endalamaw Gadisa, Endashaw Esayas, Temesgen Ashine, Desta Ejeta, Sisay Dugassa, Mekonnen Yohannes, Wossenseged Lemma, Delenasaw Yewhalaw, Sheleme Chibsa, Hiwot Teka, Matt Murphy, Melissa Yoshimizu, Dereje Dengela, Sarah Zohdy, Seth Irish

**Affiliations:** 1 Abt Associates, PMI VectorLink Ethiopia Project, Addis Ababa, Ethiopia; 2 Dire Dawa University, Dire Dawa, Ethiopia; 3 Jigjiga University, Jigjiga, Ethiopia; 4 Addis Ababa University, Addis Ababa, Ethiopia; 5 Armauer Hansen Research Institute, Addis Ababa, Ethiopia; 6 Assosa University, Assosa, Ethiopia; 7 Mekelle University, Mekelle, Ethiopia; 8 Gondar University, Gondar, Ethiopia; 9 Jimma University, Jimma, Ethiopia; 10 US President’s Malaria Initiative (PMI), Addis Ababa, Ethiopia; 11 United States Agency for International Development (USAID), Addis Ababa, Ethiopia; 12 Malaria Branch, Division of Parasitic Diseases and Malaria, Center for Global Health, Centers for Disease Control and Prevention, Atlanta, GA USA; 13 US President’s Malaria Initiative, USAID, Washington, DC USA; 14 Abt Associates, PMI VectorLink Project, Rockville, MD USA; 15 Entomology Branch Division of Parasitic Diseases and Malaria, Center for Global Health, Centers for Disease Control and Prevention, Atlanta, GA USA

## Correction to: Malar J (2021) 20:263 https://doi.org/10.1186/s12936-021–03801-3

Following publication of the article [1], it was brought to our attention that the following authors had been omitted from the author list: Desta Ejeta, Sisay Dugassa, Mekonnen Yohannes, Wossenseged Lemma^.^ These authors should be included because they organized and led the collection of the field data, and they also reviewed and approved the manuscript.

The author list has been corrected in the published manuscript and the correct author list can be found in this correction article. Likewise, the ‘Authors’ contributions’ statement has been updated in the original article and can be seen in this correction:

**Authors' contributions**

MB, SI, and DD designed the field collection protocols; MB, PM, SI, DD, FG, EG, SC, HT, MM, and MY oversaw the collection of data; MB, GY, EA, DG, SY, AW, AG, EE, TA, DE, SD, MYoh, and WL collected the field data; DY conducted the laboratory work; SI, SZ, and MB analyzed and interpreted the data; MB, SI, and SZ wrote the first draft of the manuscript. DD, MY, and GY provided a critical revision of the manuscript. All authors read and approved the final manuscript.

The authors thank you for reading this correction and apologize for any inconvenience caused.
